# Characterization of Mononucleated Human Peripheral Blood Cells

**DOI:** 10.1100/2012/843843

**Published:** 2012-05-02

**Authors:** Ruzanna Ab Kadir, Shahrul Hisham Zainal Ariffin, Rohaya Megat Abdul Wahab, Shabnam Kermani, Sahidan Senafi

**Affiliations:** ^1^School of Biosciences and Biotechnology, Faculty of Science and Technology, Universiti Kebangsaan Malaysia, 43600 Bangi, Selangor, Malaysia; ^2^Department of Orthodontics, Faculty of Dentistry, Universiti Kebangsaan Malaysia, 50300 Kuala Lumpur, Kuala Lumpur, Malaysia

## Abstract

Unspecialized cells that can renew themselves and give rise to multiple differentiated cell types are termed stem cells. The objective of this study was to characterize and investigate, through molecular and biochemical analyses, the stemness of cells derived from isolated mononucleated cells that originated from peripheral blood. The isolated mononucleated cells were separated according to their physical characteristics (adherent and suspension), after 4 to 7 days into a 14-day culturing period in complete medium. Our results revealed that adherent and suspension cells were positive for mesenchymal stem cell (MSC) and hematopoietic stem cell (HSC) markers, respectively. Differentiation of adherent cells into osteoblasts was associated with expression of the *OPN* gene and increasing ALP enzyme activity, while differentiation of suspension cells into osteoclasts was associated with expression of the *TRAP* gene and increasing TRAP enzyme activity. In conclusion, molecular and biochemical analyses showed that mononucleated cells consist of MSC (adherent) and HSC (suspension), and both cell types are able to differentiate into specialized cells from their respective lineage: osteoblast (MSC) and osteoclast (HSC).

## 1. Introduction

Stem cell research has been one of the most fascinating and controversial areas of contemporary biology. Progress in the area of stem cell research raises scientific questions as rapidly as it generates new discoveries. Stem cell research is hailed for the potential to revolutionize the future of medicine, with its ability to regenerate damaged and diseased organs. Stem cells can be defined by 3 main criteria: (1) the ability to self-renew for several cell divisions, which is a prerequisite for sustaining the stem cell pool, (2) the ability to generate at the single cell level differentiated progeny cells, generally of multiple lineages, and (3) the ability to functionally reconstitute a given tissue *in vivo* [1].

To date, several studies have shown that mesenchymal stem cells (MSCs) and hematopoietic stem cells (HSCs) are adult stem cells present in blood [2]. MSCs are generally defined as self-renewable, multipotent progenitor cells with the ability to differentiate into several mesenchymal lineages, including bone, cartilage, adipose, and muscle tissues [3]. These cells are usually identified by their plastic adherence and surface marker expression of *CD73, CD90, CD105* and absence of *CD34, CD45, HLA-DR* [4]. Meanwhile, HSCs are defined by their ability to repopulate all of the hematopoietic lineage *in vivo *and sustain the production of these cells for the lifespan of the individual [5, 6]. Several laboratories have identified HSCs as cells that demonstrate specific positive markers such as *KIT*, *Sca-1*, SLAM family markers, and lineage negativity [4, 7].

However, the method used for obtaining HSCs and MSCs from peripheral blood mononucleated cells is still debated. Isolating small amounts of MSCs detectable in peripheral blood is difficult and depends on the method used. MSCs that have been isolated are adherent and display a fibroblastic appearance [8]. Therefore, in this study, we further separated the mixed population of isolated mononucleated cells according to their physical characteristics, which are nonadherent (suspension) and adherent, after several days of culture selection.

Stemness must be addressed before a cell is to be classified as a stem cell. In this study, the stemness of isolated mononucleated cells that we have further separated according to their physical characteristics was determined through the expression of stemness markers, gene expression profiles, and biochemical analysis of adherent and suspension mononucleated cells during osteoblast and osteoclast differentiation, respectively. Our results demonstrate that adherent and suspension mononucleated cells show expression of MSC markers and HSC markers, respectively. The gene expression profiles and biochemical analyses of adherent (MSC) and suspension (HSC) mononucleated cells during *in vitro *osteoblast and osteoclast differentiation indicate that these cells are also capable of fully differentiating into osteoblast and osteoclast cells. These studies demonstrate the possibility of obtaining HSC and MSC population from mononucleated blood. Our approach may lead to the improved ability to isolate HSCs and MSCs from mononucleated peripheral blood cells and it could lead to further research in cellular therapy.

## 2. Materials and Methods

### 2.1. Isolation of Mononucleated Cells

Three samples of peripheral blood were obtained by venipuncture from each of 3 healthy volunteers (18–25 years old) after obtaining their informed consent and approval from the Ethical Committee of the Faculty of Science and Technology, Universiti Kebangsaan Malaysia (UKM). In each blood sample the cell culture experiment, gene expression, and biochemical analyses were performed. Human mononucleated peripheral blood cells were isolated using the Ficoll-Paque density-gradient separation method. Firstly, the blood samples were diluted three times with Hanks' Balanced Salt Solution (Sigma, USA). The diluted blood samples were carefully layered 1 : 1.5 on Ficoll-Paque PLUS (GE Healthcare, Sweden) and centrifuged at 400 g for 20 min at room temperature. The mononucleated cell layer at the plasma-Ficoll interface was washed 3 times with phosphate buffer saline (PBS) and cultured in 6-well plates with complete medium containing *α*-Minimal Essential Medium (*α*-MEM) (Invitrogen, USA) supplemented with 10% (v/v) newborn calf serum (NBCS) (Invitrogen, USA) and 2% (v/v) penicillin-streptomycin solution (Invitrogen, USA) at 37°C in a humidified atmosphere containing 5% CO_2_. Approximately 8 ×10^5^ mononuclear cells were isolated from 1 mL of peripheral blood samples. The isolated cells were cultured in complete medium for 14 days with complete medium exchange every 3 days. Cells were transferred into a new 6-well plate 4 to 7 days prior to separating them into plates of either adherent or suspension cells.

### 2.2. Cell Culture Morphology

The morphologies of adherent and suspension mononucleated cells were observed *in vitro* at days 0, 7, and 14 using an inverted microscope (Olympus, Model: CKX75). Images were obtained using a digital camera.

### 2.3. Differentiation of Adherent Mononucleated Cells into Osteoblasts

Approximately 1 × 10^5^ cell/mL mononucleated cells cultured in suspension in complete medium were supplemented with differentiation factors, 50 *μ*g/mL ascorbic acid (Sigma, USA) and 10 mM *β*-glycerophosphate (Sigma, USA), for 14 days to induce differentiation into osteoblasts. Cultures were maintained at 37°C with 5% CO_2_. Cells cultured in complete medium without differentiation factors were used as controls.

### 2.4. Differentiation of Suspension Mononucleated Cells into Osteoclasts

For osteoclast differentiation, 1 × 10^5^ cell/mL of suspension mononucleated cells were cultured in complete medium supplemented with growth factors, 50 ng/mL recombinant sRANKL and 25 ng/mL macrophage-colony stimulating factor (M-CSF) and cultured for 10 days. Cultures were maintained at 37°C with 5% CO_2_. Cells cultured in complete medium without growth factors were used as controls.

### 2.5. Reverse-Transcriptase Polymerase Chain Reaction (RT-PCR) Amplification

Total RNA was extracted using TRI Reagent (Sigma, USA) following the manufacturer's instructions. Total RNA was extracted from human adherent and suspension mononucleated cells that had been cultured for 14 days in complete medium, 10 and 14 days in osteoclast and osteoblast differentiation medium, respectively. One microgram total RNA was subjected to RT-PCR amplification using an Access RT-PCR System kit provided by Promega, USA. First-strand complementary DNA (cDNA) was synthesized by reverse transcription at 45°C for 45 min, followed by Avian Myeloblastosis Virus reverse transcriptase inactivation at 94°C for 2 min. Second-strand cDNA synthesis and PCR amplification consisted of 40 cycles of denaturation at 94°C for 30 sec, primer annealing for 1 min and extension at 68°C for 2 min, with a final cycle at 68°C for 7 min. The specific primer sequences used are shown in Table 1. Each RT-PCR product was then sequenced and analyzed.

### 2.6. Alkaline Phosphatase Assay

Biochemical analysis for osteoblast differentiation was conducted by Alkaline Phosphatase (ALP) enzyme assays of adherent cells cultured in osteoblast differentiation and complete medium on days 0, 3, 5, 7, 10, and 14. Adherent cells cultured in complete medium were used as the control. The cells were washed with PBS and then incubated in 0.1 M NaHCO_3_-Na_2_CO_3_ (pH 10.0), 0.1% (v/v) Triton X-100, 2 mM MgSO_4_ and 6 mM p-nitrophenyl phosphate (*ρ*NPP) (Sigma, USA) for 30 min at 37°C. The reaction was stopped by adding 1 mL 1.5 M NaOH, and absorbance was measured at 405 nm. ALP activity is represented as the specific activity. The specific activity was determined by the unit activity (U) per total protein content (mg), and the protein content was measured using the Bradford method. One unit of activity is represented by the hydrolysis of 1 *μ*M *ρ*NPP per minute at 37°C. The ALP-specific activity was presented in a percentage value, which was compared to the ALP-specific activity in the control cells (cells cultured in complete medium). ALP-specific activity in the control cells was used to determine the basal activity rate (100%).

### 2.7. Tartrate-Resistant Acid Phosphatase Assay

Biochemical analysis for osteoclast differentiation was conducted by Tartrate-Resistant Acid Phosphatase (TRAP) enzyme assays of suspension cells cultured in osteoclast differentiation and complete medium on days 0, 3, 5, 7 and 10. Suspension cells cultured in complete medium were used as the control. The cells were washed with PBS. The TRAP enzyme was assayed in cell extracts and complete medium using *ρ*NPP as a substrate in an incubation medium (1 mL) containing the following components: 10 mM *ρ*NPP, 0.1 M Na-acetate (pH 5.8), 0.15 M KCl, 0.1% (v/v) Triton X-100, 10 mM Na-tartrate, 1 mM ascorbic acid, and 0.1 mM FeCl_3_. The mixture was incubated for 1 hour and the reaction was stopped by adding 500 *μ*L of 0.3 M NaOH and immediately read at a wavelength of 405 nm. TRAP activity is expressed as the hydrolysis of 1 *μ*M pNPP per min at 37°C. The specific activity was determined by unit activity (U) per total protein content (mg), as mentioned previously. The TRAP-specific activity was presented in a percentage value, which was compared to TRAP-specific activity in the control cells (suspension cells cultured in complete medium). TRAP-specific activity in the control cells was used to determine the basal activity rate (100%).

### 2.8. Statistical Analysis

Data were statistically analyzed using paired *t*-tests. Data were considered statistically significant at *P* < 0.05.

## 3. Results

### 3.1. Morphology and Characteristics of Isolated Cells

**Figure 1:**
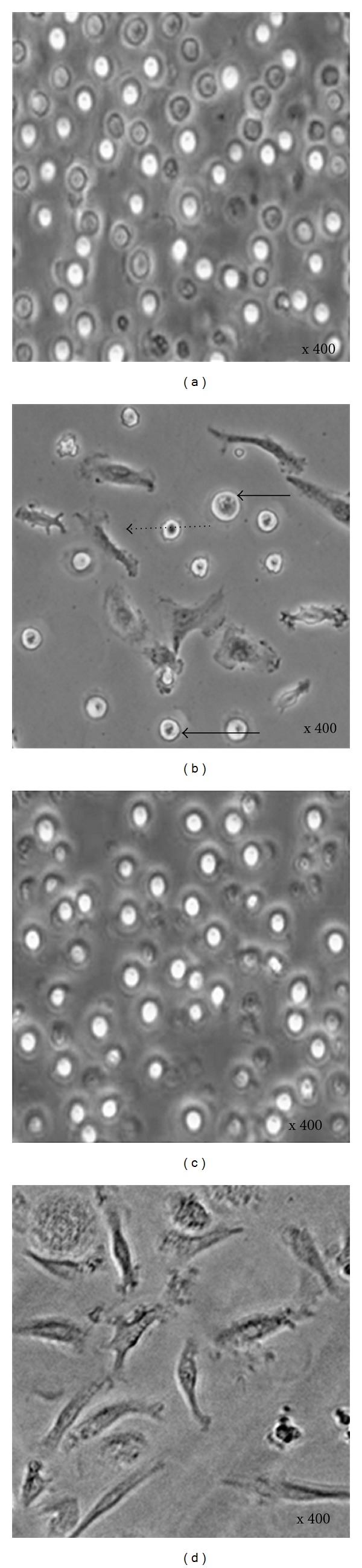
Human mononucleated cell morphology. (a) Mononucleated cells after isolation, (b) mononucleated cells after 7 days culture, (c) suspension cells, and (d) adherent mononucleated cells after 14-day culture. Each photomicrograph above is representative of three independent experiments (× 200). Cells in suspension: the solid arrow. Adherent cells: the dashed arrow.

**Figure 2:**
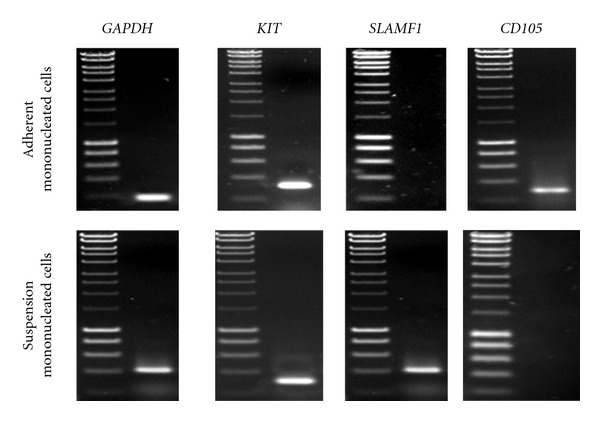
Expression of hematopoietic stem cell (HSC) and mesenchymal stem cell (MSC) markers in mononucleated cells derived from peripheral blood. RT-PCR analysis was performed using total RNA isolated from adherent and suspension mononucleated cells of human peripheral blood. *CD105 *was used as an MSC marker, *SLAM F1 *as an HSC marker, and *KIT *as a stem cell factor (SCF) marker. *GAPDH *was used as a positive control for RT-PCR analysis. A 100 bp DNA ladder was used to identify the approximate size of the RT-PCR products. The RT-PCR products were observed by electrophoresis on 1% (w/v) agarose gels and stained with ethidium bromide. All RT-PCR products were confirmed with DNA sequencing after cloning and were found to be 100% identical to the known sequences obtained from BLAST analysis.

The freshly isolated mononucleated cells from human peripheral blood (Figure 1(a)) were morphologically heterogeneous. Further culturing of these cells in complete medium for 7-day produced two different types of cells. One cell type was round and floated in the medium or loosely attached to the culture dish (suspension cells); another cell type firmly attached with a different morphological appearance (adherent cells) (Figures 1(b) and 2(b)). The adherent and suspension mononucleated cells were then separated; mononucleated cells that already attached were left to expand in the same dishes, while suspension mononucleated cells were transferred into new 6-well plates.

Adherent and suspension mononucleated cells were separated after 7 days *in vitro* culture selection. Both adherent and suspension mononucleated cells were maintained in complete medium for 14 days of culture, to deplete most of the unwanted cells and allow time for proliferation prior to enriching the cells for analysis. After 14 days in culture, the adherent and suspension cells became more morphologically homogeneous; most of the cells in suspension were morphologically round (Figure 1(c)), while the adherent mononucleated cells showed a spindle-shaped fibroblast-like morphology (Figure 1(d)). However, morphological characteristics alone are insufficient to demonstrate the presence of mononucleated stem cells. Thus, the mononucleated cells were further characterized by a molecular approach (RT-PCR analysis), using human-specific stem cell markers.

### 3.2. Expression of Mesenchymal Stem Cell (MSC) and Hematopoietic Stem Cell (HSC) Markers

**Figure 3:**
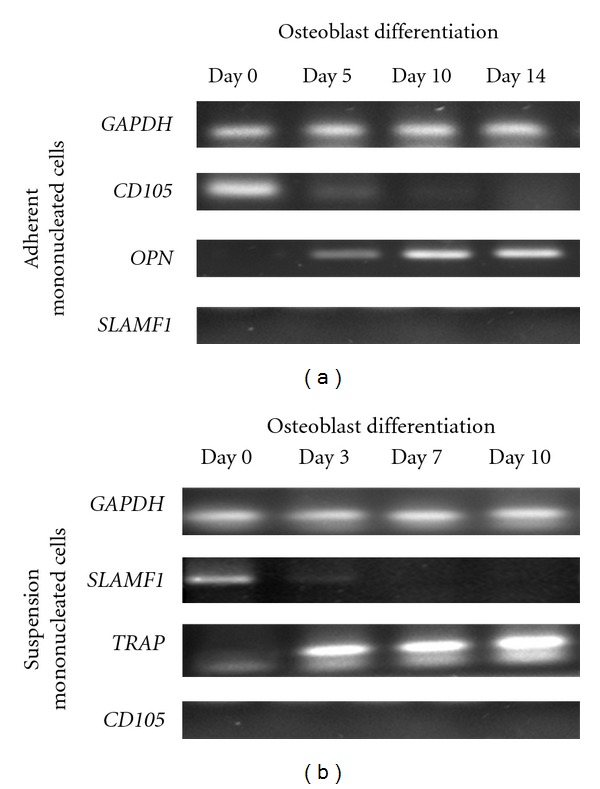
Gene expression profiles during osteoblast and osteoclast differentiation from human mononucleated cells. Adherent and suspension mononucleated cells were induced to differentiate into osteoblast (a) and osteoclast (b) cells, respectively. The expression profiles of *OPN *(234 bp) and *TRAP *(176 bp) were used as osteoblast and osteoclast markers, respectively. The expression profile of GAPDH was used as a control.

Adherent and suspension mononucleated cells from human peripheral blood (Figure 3(a)) were analyzed by RT-PCR for the presence of MSC and HSC markers. In this study, we used *CD105 *as an MSC marker, *SLAM F1 *as an HSC marker, and *KIT *as stem cell marker. Adherent and suspension mononucleated cells (Figure 2) were shown to express MSC and HSC markers, respectively. Furthermore, adherent and suspension mononucleated cells were negative for the presence of HSC and MSC markers, respectively. RT-PCR showed amplification of products of the expected size for the MSC marker: *CD105* (290 bp), HSC marker: *SLAM F1* (403 bp), and stem cell marker: *KIT* (316 bp).

### 3.3. Gene Expression Profiles during Osteoblast and Osteoclast Differentiation

Gene expression profiles of adherent and suspension mononucleated cells during osteoblast and osteoclast differentiation were carried out to evaluate the capacities of MSCs from adherent and HSCs from suspension mononucleated cells to differentiate into specialized cells from their expected lineage. RT-PCR was performed to examine how gene expression of MSC, HSC, osteoblast and osteoclast marker profiles changes during osteoblast and osteoclast differentiation. RT-PCR analyses were conducted simultaneously at days 0, 5, 10, and 14 for osteoblast differentiation and at days 0, 3, 7, and 10 for osteoclast differentiation. RT-PCR analyses showed different levels of gene expression in (Figure 3) mononucleated cells during osteoblast and osteoclast differentiation. The markers for MSCs (*CD105*), HSCs (*SLAM F1*), osteoblasts (*OPN*), and osteoclasts (*TRAP*) were used. As seen in Figure 3(a), the expression of the osteoblast marker was low during early osteoblast differentiation but increased over time, similar to osteoclast differentiation of mononucleated cells and increased osteoclast marker expression throughout the differentiation period. The increases in osteoblast and osteoclast marker transcription suggest that the mononucleated cells differentiated into mature osteoblast and osteoclast cells, respectively. Moreover, the expression of stemness markers in both adherent and suspension suspension mononucleated cells decreased over the time in differentiation media, which indicates that the unspecialized mononucleated stem cells have successfully differentiated.

### 3.4. Biochemical Analysis during Osteoblast and Osteoclast Differentiation

**Figure 4:**
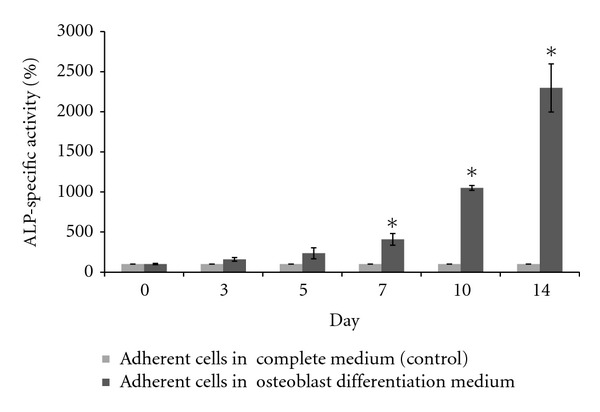
Percentage of ALP-specific activity of adherent mononucleated cells during osteoblast differentiation. There was significantly increased ALP activity in adherent cells cultured in osteoblast differentiation medium at days 7, 10, and 14 of differentiation, using a paired *t*-test *indicates a significant (*P* < 0.05) increase in ALP enzyme activity compared to control cells. Results are presented as the mean ± SD (*n* = 3). ALP-specific activity in control cells was used for the basal rate (100%).

Biochemical analyses during osteoblast and osteoclast differentiation were carried out by two different enzyme assays: ALP and TRAP assays. ALP is a relative marker of osteoblast differentiation, while TRAP is a biochemical marker for osteoclasts.  Figure 4 shows the percentage of ALP-specific activity during 14 days of osteoblast differentiation. The percentage of ALP-specific activities in adherent mononucleated cells cultured in osteoblast differentiation medium gradually increased from day 3 to day 14 of osteoblast differentiation. This was statistically significant (*P* < 0.05) starting from days 7, 10, and 14 of osteoblast differentiation (Figure 4). Therefore, adherent cells, which consist of MSCs, were successfully differentiated into osteoblasts.

**Figure 5:**
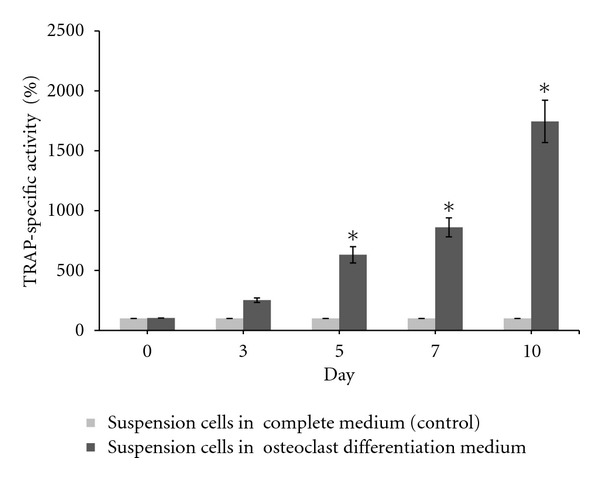
Percentage of TRAP-specific activity of suspension mononucleated cells during osteoclast differentiation. There was a significant increase in TRAP activity in adherent cells cultured in osteoblast differentiation medium at day 5, 7, and 10 of differentiation, using a paired *t*-test *indicates a significant (*P* < 0.05) increase in TRAP enzyme activity compared to control cells. Results are presented as the mean ± SD (*n* = 3). TRAP-specific activity in control cells was used for the basal rate (100%).

Meanwhile, biochemical analysis using TRAP enzyme assays during osteoclast differentiation showed that the percentage of TRAP-specific activities increased in suspension mononucleated cells cultured in osteoclast differentiation medium starting from day 3 to day 10 of the induction of differentiation (Figure 5). Statistical analysis using a paired *t*-test for TRAP enzyme assays showed a significant increase (*P* < 0.05) at days 5, 7, and 10 of differentiation. These results indicate that the suspension cells were successfully differentiated into osteoclasts, as TRAP is used as a biochemical marker for osteoclasts.

## 4. Discussion

The isolation of mononucleated cells was based on density gradient centrifugation using Ficoll-Paque, in which differences in density separate mononucleated cells from other blood cells. As shown in Figure 1(a), the isolated mononucleated cells are in a heterogeneous population. After 7 days of culture in complete medium, we obtained a mixed population with a low number of fibroblast-like cells (adherent) and suspension cells that have one round nucleus (e.g., lymphocytes, monocytes, and macrophages) (Figure 1(b)). Both adherent and suspension cells were cultured separately for additional 7 days. After a total of 14 days of *in vitro* culture selection, the adherent and suspension cells became more morphologically homogenous (Figures 1(c) and 1(d)) as we suspect that a majority of differentiated and precursor cells died due to their short lifespan, for example, granulocytes (30–40 minutes in the peripheral blood with a total lifespan of 7–13 days that varied under certain pathological conditions), monocytes (5–7 days), and platelets (3–5 days) [9].

For a particular cell type to be classified as a stem cell, proof of stemness through proper and accepted characterization tests must be addressed. Stemness refers to the stem cell properties of having a certain gene or genes whose expression makes possible both the potential for self-renewal and multilineage differentiation [10]. In our study, *CD105 *was used as an MSC marker, *SLAM F1 *as an HSC marker, and *KIT *as stem cell marker (Figure 3(a)). KIT or c-Kit or CD117 is a Stem Cell Factor (SCF) receptor and expression of *KIT* in both types of stem cells is important as it acts on migration, adhesion, survival, proliferation, and differentiation [11]. *KIT* expression was reported in adult bone marrow hematopoietic and mesenchymal stem cells [12, 13] as well as fetal and umbilical cord hematopoietic stem cells [14, 15]. According to Hassan [16], its expression correlates with the self-renewal function of these fetal and adult stem cells. Moreover, *KIT* was found expressed in both human and mouse undifferentiated embryonic stem cells with a role in maintaining their undifferentiated state and correlation with functional measures of their pluripotency [17–21]. *CD105* or endoglin is a type I membrane glycoprotein, which is located on the cell surface and is also part of the TGF-*β* receptor complex [22]. *CD105* is involved in regulating the proliferation of MSCs [23]. *SLAMF1* is a cell surface receptor important in the self-renewal of HSCs [24] and is the founding member of the SLAM family of cell surface receptors [25, 26]. SLAM family members regulate the proliferation and activation of lymphocytes [27, 28].

This study revealed that adherent cells were positive for the expression of MSC markers, with an absence of HSC marker expression. By contrast, suspension cells were positive for the presence of HSC markers, while MSC marker expression was absent (Figure 2). These data indicate that adherent mononucleated cells with fibroblast-like morphology are MSCs, while HSCs are suspension mononucleated cells with round nuclear morphology.

The ability to generate differentiated progeny is one stem cell property (stemness). The stemness of adherent and suspension mononucleated cells was further determined by gene expression profiles and biochemical analysis during osteoblast and osteoclast differentiation. MSCs have the ability to differentiate into all of the mesenchymal lineages, while HSCs have the ability to repopulate the hematopoietic lineages. Thus, adherent cells (MSCs) were differentiated into osteoblast cells that originated from mesenchymal lineages, while suspension cells (HSCs) were induced to differentiate into osteoclast cells that originated from hematopoietic lineages.

Gene expression profiles demonstrated that the stemness markers were downregulated upon the induction of differentiation, resulting in decreased marker expression. All of the stemness markers, such as *CD105* and *SLAM F1,* which have been used to examine gene expression profiles, play a significant role in the maintenance of the undifferentiated state of cells by stimulating proliferation [23, 24, 29]. Therefore, the downregulated of these genes might play important roles in the initiation of the differentiation process in mononucleated cells. By contrast, osteoblast and osteoclast markers were upregulated during differentiation, which resulted in the increased expression of these markers.

*ALP* [30], osteopontin (*OPN*), and osteocalcin (*OC*) [31] genes have been widely used as osteoblast markers, while cathepsin K (*CATK*) and *TRAP* are used as osteoclast markers [32]. The increase in *OPN* gene transcription (Figure 3(a)) shows that mononucleated cells differentiate into mature osteoblast cells [9], while increased expression of *TRAP* (Figure 3(b)) indicates that mononucleated cells have differentiated into osteoclast cells [33, 34]. The housekeeping gene (*GAPDH*) is constitutively expressed at the same level throughout differentiation. This finding is in agreement with previous findings by Barber et al. [35], which showed that *GAPDH* is constitutively expressed at a relatively constant level in mammalian cells or tissues (Figure 3).

Biochemical analysis was performed to further confirm that adherent and suspension mononucleated cells were able to differentiate into osteoblast and osteoclast cells, respectively, when exposed to the appropriate induction medium. In addition to the increased expression of *OPN*, osteoblasts also actively produce the ALP enzyme [32]. Therefore, ALP enzyme activity assays were carried out to detect the presence of active osteoblast cells, when adherent cells were cultured in the differentiation osteoblast medium that contains ascorbic acid and *β*-glycerophosphate. As seen in Figure 4, ALP enzyme activity was significantly higher from day 7 until day 14 of osteoblast differentiation in the presence of these two differentiation factors. This finding is in agreement with previous findings in which Zainal Ariffin et al. [9], Zainol Abidin et al. [33, 34], and Yazid et al. [36] showed that ALP activity increased during osteoblast differentiation. The ALP enzyme is associated with bone formation of osteoblast cells [37] as it is involved in producing high levels of local phosphate ions that provide the chemical conditions needed for mineral deposition, which induces the mineralization of osteoblast cells [38].

TRAP enzyme activity assays were carried out to detect the presence of osteoclast cells when suspension cells were cultured in the differentiation osteoclast medium, which contains RANKL and M-CSF. TRAP enzymes were secreted at the highest levels at day 10 of osteoclast differentiation (Figure 5). These data are also in accord with the findings of Zainal Ariffin et al. [9], Zainol Abidin et al. [33, 34], and Yazid et al. [36], who reported that TRAP enzyme activity was highest at day 10 of osteoclast differentiation from mouse cells in suspension. Furthermore, the increased activity of TRAP enzymes was concurrent with increased expression of the *TRAP *gene in the gene expression profile analyses. The adherent and suspension mononucleated cells, therefore, had the capacity to differentiate into osteoclasts and osteoblasts, respectively, when exposed to the appropriate induction medium. Thus, in this study, the gene expression profile and biochemical analyses of mononucleated cells during differentiation revealed that both adherent and suspension mononucleated cells were successfully differentiated into more specialized cells.

## 5. Conclusions

Using the expression of stem cell markers, adherent and suspension mononucleated cells were shown to have MSC and HSC characteristics, respectively. Observation of the gene expression profiles and biochemical analyses of adherent and suspension mononucleated cells during osteoblast and osteoclast differentiation, respectively, indicates that these cells are also capable of fully differentiating into their respective cell types. Therefore, our isolation procedure could be useful as the first step in isolating more homogeneous populations of MSCs and HSCs, which are essential for functional and molecular investigations into mechanisms that regulate their self-renewal, lineage restriction, and differentiation.

## Figures and Tables

**Table 1:** Human primer sequences used in RT-PCR.

Gene	Primer	Sequence	Annealing temperature (°C)	Expected size (bp)
*GAPDH*	Sense	5′CCATGGAGAAGGCTGG3′	55	195
	Antisense	5′CAAAGTTGTCAGGATGACC3′		
*SLAMF1*	Sense	5′CTCTGCGTTCTGCTCCTA3′C	54	403
	Antisense	5′TTGGTCACTTCTGGGTCTG3′		
*KIT*	Sense	5′TCCTTGACCTTCGTGCTGT3′	53	316
	Antisense	5′CCTTCCTGCTTTCCCGTAT3′		
*CD105*	Sense	5′GCTCCCTCTGGCTGTTG3′	61	290
	Antisense	5′TTACACTGAGGACCAGAAGC3′		
*TRAP*	Sense	5′GACCACCTTGGCAATGTCTCTG3′	58	176
	Antisense	5′TGGCTGAGGAAGTCATCTGAGTTG3′		
*OPN*	Sense	5′GACCTGCCAGCAACCGAAGT3′	56	452
	Antisense	5′GGGTCTACAACCAGCATA3′		

## References

[1] Weissman IL (2000). Translating stem and progenitor cell biology to the clinic: barriers and opportunities. *Science*.

[2] Yazid MD, Zainal Ariffin SH, Senafi S, Zainal Ariffin Z, Wahab RMA (2011). Stem cell heterogeneity of mononucleated cells from murine peripheral blood: molecular analysis. *The Scientific World Journal*.

[3] Alhadlaq A, Mao JJ (2004). Mesenchymal stem cells: isolation and therapeutics. *Stem Cells and Development*.

[4] Tögel F, Westenfelder C (2007). Adult bone marrow-derived stem cells for organ regeneration and repair. *Developmental Dynamics*.

[5] Zainal Ariffin SH, Wahab RMA, Zainol Abidin IZ, Senafi S, Nor Muhammad M, Zaidah ZA (2005). Stem Cell in Blood Development. *Sains Malaysiana*.

[6] Wognum AW, Eaves AC, Thomas TE (2003). Identification and isolation of hematopoietic stem cells. *Archives of Medical Research*.

[7] Kiel MJ, Yilmaz ÖH, Iwashita T, Yilmaz OH, Terhorst C, Morrison SJ (2005). SLAM family receptors distinguish hematopoietic stem and progenitor cells and reveal endothelial niches for stem cells. *Cell*.

[8] Bobis S, Jarocha D, Majka M (2006). Mesenchymal stem cells: characteristics and clinical applications. *Folia Histochemica et Cytobiologica*.

[9] Zainal Ariffin SH, Zainol Abidin IZ, Yazid MD, Wahab RMA (2010). Differentiation analyses of adult suspension mononucleated peripheral blood cells of Mus musculus. *Cell Communication and Signaling*.

[10] Leychkis Y, Munzer SR, Richardson JL (2009). What is stemness?. *Studies in History and Philosophy of Science Part C*.

[11] Nakamura Y, Tajima F, Ishiga K (2004). Soluble c-kit receptor mobilizes hematopoietic stem cells to peripheral blood in mice. *Experimental Hematology*.

[12] Hassan HT, El-Sheemy M (2004). Adult bone-marrow stem cells and their potential in medicine. *Journal of the Royal Society of Medicine*.

[13] Majumdar MK, Thiede MA, Haynesworth SE, Bruder SP, Gerson SL (2000). Cutting edge communication: human marrow-derived mesenchymal stem cells (MSCs) express hematopoietic cytokines and support long-term hematopoiesis when differentiated toward stromal and osteogenic lineages. *Journal of Hematotherapy and Stem Cell Research*.

[14] Bowie MB, Kent DG, Copley MR, Eaves CJ (2007). Steel factor responsiveness regulates the high self-renewal phenotype of fetal hematopoietic stem cells. *Blood*.

[15] He X, Gonzalez V, Tsang A, Thompson J, Tsang TC, Harris DT (2005). Differential gene expression profiling of CD34^+^ CD133^+^ umbilical cord blood hematopoietic stem progenitor cells. *Stem Cells and Development*.

[16] Hassan HT (2009). c-Kit expression in human normal and malignant stem cells prognostic and therapeutic implications. *Leukemia Research*.

[17] Palmqvist L, Glover CH, Hsu L (2005). Correlation of murine embryonic stem cell gene expression profiles with functional measures of pluripotency. *Stem Cells*.

[18] Lu M, Glover CH, Tien AH, Humphries RK, Piret JM, Helgason CD (2007). Involvement of tyrosine kinase signaling in maintaining murine embryonic stem cell functionality. *Experimental Hematology*.

[19] Sperger JM, Chen X, Draper JS (2003). Gene expression patterns in human embryonic stem cells and human pluripotent germ cell tumors. *Proceedings of the National Academy of Sciences of the United States of America*.

[20] Chambers I, Smith A (2004). Self-renewal of teratocarcinoma and embryonic stem cells. *Oncogene*.

[21] Bashamboo A, Taylor AH, Samuel K, Panthier JJ, Whetton AD, Forrester LM (2006). The survival of differentiating embryonic stem cells is dependent on the SCF-KIT pathway. *Journal of Cell Science*.

[22] Duff SE, Li C, Garland JM, Kumar S (2003). CD105 is important for angiogenesis: evidence and potential applications. *The FASEB Journal*.

[23] Gaebel R, Furlani D, Sorg H (2011). Cell origin of human mesenchymal stem cells determines a different healing performance in cardiac regeneration. *PLoS One*.

[24] Kent DG, Copley MR, Benz C (2009). Prospective isolation and molecular characterization of hematopoietic stem cells with durable self-renewal potential. *Blood*.

[25] Engel P, Eck MJ, Terhorst C (2003). The SAP and SLAM families in immune responses and X-linked lymphoproliferative disease. *Nature Reviews Immunology*.

[26] Sidorenko SP, Clark EA (2003). The dual-function CD 150 receptor subfamily: the viral attraction. *Nature Immunology*.

[27] Howie D, Okamoto S, Rietdijk S (2002). The role of SAP in murine CD150 (SLAM)-mediated T-cell proliferation and interferon *γ* production. *Blood*.

[28] Wang N, Satoskar A, Faubion W (2004). The cell surface receptor SLAM controls T cell and macrophage functions. *Journal of Experimental Medicine*.

[29] Bruzzone S, de Flora A, Usai C, Graeff R, Lee HC (2003). Cyclic ADP-ribose is a second messenger in the lipopolysaccharide-stimulated proliferation of human peripheral blood mononuclear cells. *Biochemical Journal*.

[30] Asma AAA, Wahab RMA, Zainal Ariffin SH (2008). Crevicular alkaline phosphatase activity during orthodontic tooth movement: canine retraction stage. *Journal of Medical Sciences*.

[31] D’Alonzo RC, Kowalski AJ, Denhardt DT, Nickols GA, Partridge NC (2002). Regulation of collagenase-3 and osteocalcin gene expression by collagen and osteopontin in differentiating MC3T3-E1 cells. *The Journal of Biological Chemistry*.

[32] Kartsogiannis V, Ng KW (2004). Cell lines and primary cell cultures in the study of bone cell biology. *Molecular and Cellular Endocrinology*.

[33] Zainol Abidin IZ, Zainal Ariffin SH, Wahab RMA, Sahidan S, Zaidah ZA (2008). Osteoclast and osteoblast development of Mus musculus haemopoietic mononucleated cells. *Journal of Biological Sciences*.

[34] Zainol Abidin IZ, Zainal Ariffin SH, Ariffin ZZ, Wahab RMA (2010). Potential differentiation of three types of primitive cells originated from different proliferation term of mouse blood. *Sains Malaysiana*.

[35] Barber RD, Harmer DW, Coleman RA, Clark BJ (2005). GAPDH as a housekeeping gene: analysis of GAPDH mRNA expression in a panel of 72 human tissues. *Physiological Genomics*.

[36] Yazid MD, Zainal Ariffin SH, Senafi S, Razak MA, Wahab RMA (2010). Determination of the differentiation capacities of murines’primary mononucleated cells and MC3T3-E1 cells. *Cancer Cell International*.

[37] Zainal Ariffin SH, Yamamoto Z, Zainol Abidin IZ, Wahab RMA, Zainal Ariffin Z (2011). Cellular and molecular changes in orthodontic tooth movement. *The Scientific World Journal*.

[38] Coelho MJ, Fernandes MH (2000). Human bone cell cultures in biocompatibility testing. Part II: effect of ascorbic acid, *β*-glycerophosphate and dexamethasone on osteoblastic differentiation. *Biomaterials*.

